# Mechanical Determinants of the U-Shaped Speed-Energy Cost of Running Relationship

**DOI:** 10.3389/fphys.2018.01790

**Published:** 2018-12-18

**Authors:** Apolline Carrard, Elisa Fontana, Davide Malatesta

**Affiliations:** Faculty of Biology and Medicine, Institute of Sport Sciences, University of Lausanne, Lausanne, Switzerland

**Keywords:** biomechanics, optimal speed, hypogravity, stretch-shortening cycle, elastic energy

## Abstract

**Purpose:** The aim of this study was to investigate the relationship between the energy cost of running (Cr) and speed and its mechanical determinants by comparing running in normal [100% body weight (BW)] and reduced (20% and 60% BW) gravity conditions at several speeds (2.25, 3.17, 4.08, and 5.00 m·s^−1^) in experienced runners.

**Methods:** Twelve experienced runners (24.6 ± 5.4 year) ran on an *AlterG* treadmill in a partially randomized order at the four running speeds and at the three gravity conditions in order to assess Cr, spatiotemporal parameters, spring-mass characteristics and elastic energy (EL) during running.

**Results:** For the three gravity conditions, the speed-Cr per kg of body mass relationship was curvilinear (significant speed effect: *P* < 0.001) and was significantly downward shifted with reduced gravity (100%>60%>20% BW; *P* < 0.001). EL, expressed in J·step^−1^, was significantly higher at 100% BW than at 60 and 20% BW and at 60% BW than at 20% BW (significant gravity effect: *P* < 0.001) with a significant increase in EL per step at faster speeds for the 3 gravity conditions (*P* < 0.001). EL, expressed in J·kg^−1^·m^−1^, was significantly downward shifted with gravity (100%>60%>20% BW; *P* < 0.001), with no significant speed effect (*P* = 0.39).

**Conclusions:** Our findings showed that, for the three gravity conditions, the speed-Cr relationship was curvilinear, and the optimization of the stretch-shortening cycle and muscle activation in the muscle-tendon unit may be involved to explain these U-shaped relationships, especially at normal terrestrial gravitational conditions (100% BW). The U-shaped speed-Cr per kg of the body mass relationship was shifted downward in hypogravity conditions, which was characterized by decreased EL compared to 100% BW. These mechanisms may contribute to the less than proportional decrease in Cr per kg of body mass relative to gravity.

## Introduction

The energy cost of running (Cr) is the energy demand per unit distance normalized to body mass and represents an assessment of running economy. Cr is one of the physiological determinants of distance running performance (see Foster and Lucia, 2007 for review) and discriminates between performances in athletes with similar maximal oxygen uptake (Bassett and Howley, 2000). Historically, the relationship between Cr and running speed has been described as linear, thus Cr is independent of speed (see Kram and Taylor, 1990; Bramble and Lieberman, 2004 for review). However, recent evidence has shown that Cr is not independent of running speed, and the speed-Cr relationship follows a U-shaped curve (Steudel-Numbers et al., 2007; Fletcher et al., 2009; Steudel-Numbers and Wall-Scheffler, 2009; Willcockson and Wall-Scheffler, 2012; Shaw et al., 2013; Rathkey and Wall-Scheffler, 2017; Black et al., 2018), showing a minimum for an optimal speed of ~3.5 m·s^−1^ (~12.6 km·h^−1^) (Steudel-Numbers et al., 2007; Steudel-Numbers and Wall-Scheffler, 2009; Willcockson and Wall-Scheffler, 2012; Rathkey and Wall-Scheffler, 2017; Black et al., 2018). These conflicting findings may in part be due to the small sample of runners, relatively small range of speeds (i.e. ≤ 1.1 m·s^−1^ or 4 km·h^−1^) and differences in absolute speeds used in previous studies, which may have limited their ability to describe the full speed-Cr relationship (Black et al., 2018).

The mechanisms potentially involved in this curvilinear relationship and in the optimal running speed remain unclear and controversial. Willcockson and Wall-Scheffler (2012) showed that the locomotor-respiratory coupling (entrainment) is not a determinant of optimal running speed. Therefore, they suggested that the storage and release of the elastic energy during running might be implicated to explain this energetically optimal running speed and the U-shaped speed-Cr relationship. In fact, during the eccentric phase of the ground contact, the elastic energy is stored, then returned during the concentric phase of the movement in the “elastic elements” of the muscle-tendon unit [stretch-shortening cycle (SSC)], reducing the metabolic cost of running (Cavagna et al., 1964). In other words, the lower limbs can be considered as springs loaded by the mass of the runner (i.e., the spring-mass model; Cavagna et al., 1964; Blickhan, 1989; McMahon and Cheng, 1990). Many authors have shown the reliability of the spring-mass model for describing and predicting the mechanics of running in humans (McMahon and Cheng, 1990; Farley and González, 1996). The potential benefit of this model is to integrate and include all the complex structures and phenomena (neuromuscular, tendinous and articular), which are the basis of running under a limited number of parameters (Farley and Ferris, 1998). Moreover, the elastic property of the linear lower limb spring is characterized by stiffness, which is defined as the ratio between the stretching force applied to muscle-tendon units and their lengthening. During running, the changes in the stiffness of the spring, which can be described as the leg stiffness [k_leg_; the ratio between maximal ground reaction force (F_max_) and lower limb deformation (ΔL)] and vertical stiffness [k_vert_; the ratio between F_max_ and vertical displacement of the center of mass (Δy)], may induce a modification of the storage-release of elastic energy (EL) (Saibene and Minetti, 2003). In fact, at slow running speeds, the storage and return of EL may be reduced compared to fast speeds because the lower limb adjustment to maintain a stable bouncing gait becomes more critical and requires more neuromotor control from muscles fibers (Seyfarth et al., 2002; Sasaki and Neptune, 2006), inducing an increased metabolic cost of running (Biewener and Roberts, 2000; Sasaki and Neptune, 2006). The storage-release of EL becomes greater with fast running speeds (Lai et al., 2014). However, with faster speeds, this increased storage-release of EL seems to occur at the expense of muscle fibers which function under less favorable contractile conditions (i.e., the muscle fibers operating regions shifted down the ascending limb of the force-length relationship; Lai et al., 2014). To counter these increasing unfavorable contractile conditions, a greater volume of active muscle recruited is needed and results in an increased metabolic cost of running at these speeds (Lai et al., 2014; Kipp et al., 2018). In addition, the higher EL values with increasing running speeds have been found when EL was expressed in J·step^−1^. However, as step length increases with speed, EL, when expressed in J·kg^−1^·m^−1^ to be properly compared to Cr also expressed in J·kg^−1^·m^−1^, could be also reduced at fast running speeds. Therefore, the non-optimal storage and return of EL (J·kg^−1^·m^−1^) at slow and fast running speeds and the greater muscle activation at fast running speeds may contribute to increasing the energy cost at the extremities of the speed-Cr relationship, making the latter curvilinear.

However, recently, Fletcher and MacIntosh (2015) brought into question the relevance of tendon strain and energy return alone in reducing energy cost of running and suggested an alternative mechanism. An optimal tendon stiffness would minimize muscle fascicle shortening and, thus, the required level of activation for a given force and, consequently, the energy cost during steady-state submaximal running. Although these findings are novel and relevant, they result from indirect inferences assessed at the *triceps surae* and Achilles tendon level. Further, the *triceps surae* and Achilles tendon are not the only muscle-tendon unit recruited during running. Similar to Achilles tendon, the mechanical properties of the patellar tendon, related to tendon spring-like function, are involved in the optimization of the utilization of elastic energy due to activity-driven adaptations (Wiesinger et al., 2016, 2017). Therefore, to understand the energetics of running and the role of muscle-tendon unit, we need more studies measuring all relevant parameters *in vivo* and in dynamic conditions and not only by indirect inferences. Hence, the use of the spring-mass model, to globally assess the lower limb “active” stiffness and storage-release of EL per unit distance during running for a wide range of speeds, seems rational and relevant to investigate the mechanical determinants of the U-shaped speed-Cr relationship *in vivo*.

Recently, Black et al. (2018) showed that a wide range of running speeds is needed to properly describe the U-shaped speed-Cr relationship. If only well-trained endurance runners are tested, the energy supply remains predominantly aerobic, even at faster speeds, and indirect calorimetry appears suitable for assessing energy consumption. Another option to decrease the anaerobic energy contribution at fast running speeds and concomitantly modify the storage and release of EL during running is to assess Cr in experienced runners in reduced gravity (Grabowski and Kram, 2008; Gojanovic et al., 2012). In fact, in reduced gravity, contact time (t_c_), Δy and ΔL are decreased compared to running in normal gravity at the same speed (He et al., 1991; Donelan and Kram, 2000; Pavei et al., 2015). These modifications induce a change in the mechanical properties of the muscle-tendon unit, with an increase in k_vert_ and a decrease in k_leg_ compared to that under normal gravity (He et al., 1991; Donelan and Kram, 2000; Pavei et al., 2015), likely causing decreased EL in hypogravity. This may contribute to the disproportional decrease in Cr with respect to the reduction in the transported body weight (BW) under reduced gravity (Teunissen et al., 2007; Grabowski et al., 2010; Raffalt et al., 2013). However, no studies have investigated the change of Cr as a function of running speed under conditions of reduced gravity (linear vs. curvilinear relationship) and its mechanical determinants compared to normal gravity.

Therefore, the aim of this study was to investigate the relationship between Cr and running speed and its mechanical determinants comparing running under normal (100% BW) and reduced (20 and 60% BW) gravity conditions at several speeds (2.25, 3.17, 4.08, and 5.00 m·s^−1^) with experienced runners. We hypothesized that: (1) under normal gravity conditions (100% BW), the speed-Cr relationship would be curvilinear because at the slowest speed (2.25 m·s^−1^), and at the fastest speed (5.00 m·s^−1^), EL storage per unit distance would be reduced and may induce an increase in Cr at these speeds; (2) under reduced gravity conditions (20 and 60% BW), these relationships would also be curvilinear but downward shifted compared to that at 100% (decrease in the Cr according to the BW carried) but with a reduced contribution of SSC due to the reduced gravity (i.e., EL and k_leg_ lower when gravity decreases).

## Methods

### Participants

Twelve healthy male endurance athletes (runners, triathletes, and cross-country skiers) [24.6 ± 5.4 year; 1.79 ± 0.06 m; lower limb length (the great trochanter-to-ground distance in a standing position): 96 ± 5 cm; 70.1 ± 5.1 kg; personal best record for running 10 km: 35.6 ± 2.4 min] volunteered and gave written informed consent to participate in this study. This had been approved by the local ethics committee (Cantonal Swiss Ethics Committees on research involving humans). All participants were regular runners and the main criterion to take part in this study was to be able to comfortably run 10 km ≤40 min.

### Experimental Design

Participants visited the laboratory on two occasions (familiarization and experimental sessions) wearing the same running shoes. In the familiarization session, participants' anthropometric assessments and personal and training information were collected. Different running speeds (2.25, 3.17, 4.08, and 5.00 m·s^−1^) and different gravity conditions (20, 60, and 100% BW) were tested on a treadmill that can simulate anti-gravity conditions (AlterG® Anti-Gravity Treadmill® Pro 200, Fremont, USA). Each participant experienced 12 conditions (4 speeds and 3 gravity conditions) for 3 min. The same order was applied for all participants who were not aware of the conditions under which they were running.

In the experimental session, the body mass was measured and the order of the experimental conditions was partially randomized for all participants who were not aware of the condition under which they were running. The randomization had 3 particularities: (1) no experimental trial started with one of the two fastest speeds (4.08 or 5.00 m·s^−1^); (2) these fast speeds never followed each other; and (3) the first condition of gravity was 100% BW for each participant. The experimental trial started with 5 min at rest in standing position to collect metabolic data and consisted of a minimum of 5 min of running at each speed (2.25, 3.17, 4.08, and 5.00 m·s^−1^) and gravity condition (20, 60, and 100% BW), interspersed by 5 min rest periods. For each experimental condition, the metabolic and biomechanical data (20 consecutive steps) were collected.

### Assessments

All assessments were performed during running on an AlterG® Anti-Gravity Treadmill®. This is an enclosed treadmill body-weight support system that uses a small increase in air pressure around the user's lower body to create a lifting force approximately at the person's center of mass. Each participant wore a pair of flexible neoprene shorts that included a kayak-style spray skirt and zipper that attached to the aperture (Grabowski and Kram, 2008) to guarantee the hermeticity. The runner was free to move in all directions, without restriction, and we instructed the participants to run in the middle of the AlterG® chamber aperture to minimize the horizontal assistance (Grabowski and Kram, 2008).

#### Energy Cost of Running

Oxygen uptake ($\overset{\cdot }{V}$O_2_, ml·min^−1^ or mlO_2_·kg^−1^·min^−1^), CO_2_ output ($\overset{\cdot }{V}$CO_2_, ml·min^−1^), respiratory exchange ratio (RER) and ventilation were measured breath-by-breath (Oxycon Pro^TM^, CareFusion, San Diego, USA). Before each experimental trial, the metabolic cart was calibrated with 16% O_2_ and 5% CO_2_ at low, medium and high flow rates utilizing a 3-l air syringe, according to the manufacturer's recommendations. During running trials, the experimenters visually determined when the steady state of $\overset{\cdot }{V}$O_2_ and $\overset{\cdot }{V}$CO_2_ was reached for each running speed and for each participant. For this reason, some of the participants ran more than 5 min (minimum trial duration): 5.00 ± 0.00, 5.03 ± 0.05, 5.25 ± 0.08, and 6.5 ± 0.73 min at 2.25, 3.17, 4.08, and 5.00 m·s^−1^, respectively. Afterwards, an objective assessment of steady state of $\overset{\cdot }{V}$O_2_ was performed calculating the slope of the linear regression between $\overset{\cdot }{V}$O_2_ and time during the last minute of running for each speed and runner (please see the “Statistical analysis” paragraph). During steady state (i.e., the last minute of running), the RER was lower than 1.0 (i.e., the oxidative metabolism was the main metabolic pathway) for all participants and running conditions. Breath-by-breath $\overset{\cdot }{V}$O_2_ data were initially examined to exclude errant breaths due to coughing or swallowing, and those values lying more than 3 standard deviations (SD) from the local mean were deleted. Subsequently, $\overset{\cdot }{V}$O_2_ values from the last minute were averaged and normalized to the body mass and converted to gross metabolic rate using a standard equation (Astrand and Rodahl, 1986). Then, this latter value was divided by the running speed to obtain the energy cost of running (Cr, J·kg^−1^·m^−1^). For 100, 60, and 20% BW, Cr was divided by gravity (1, 0.6, and 0.2 *g*, respectively) to obtain Cr per body weight (J·N^−1^·m^−1^; “the cost of force generation” Taylor, 1985). Both linear (y = ax+b) and second-order (y = ax^2^+bx+c) least squares regressions were used to model the relationships between Cr (J·kg^−1^·m^−1^) and running speed. Then, using the speed-Cr curvilinear relationships, the optimal running speed (i.e., the speed at which Cr is the lowest) was calculated for each participant for the 3 gravity conditions.

As previously suggested (Fletcher et al., 2009) and for sake of clarity, we prefer to report only the gross Cr, instead of the net Cr (i.e., the difference between the steady-state $\overset{\cdot }{V}$O_2_ and resting $\overset{\cdot }{V}$O_2_ divided by running speed), because (1) it can not be confirmed that resting $\overset{\cdot }{V}$O_2_ persists at the same rate during running (Stainsby and Barclay, 1970); and (2) minimizing the gross Cr is a much better predictor of the speeds at which individuals prefer to move (Srinivasan, 2009). Moreover, the results of net Cr substantially confirm those of the gross Cr reported in the Results section of this manuscript (see the Additional File in Supplementary Material for the specific results of the net Cr).

#### Spatiotemporal Parameters

For each experimental condition and after 4.5 min of running, the t_c_, flight time (t_f_), step frequency and length were assessed during 20 consecutive steps by short video sequences (15 s) with a digital camera HERO4 black® (GoPro Inc., San Mateo, CA, USA) recording at 240 Hz and with dedicated software for subsequent analysis (Dartfish, Fribourg, Suisse). To improve the image quality, a lamp was placed inside the treadmill (LED-312, Vidpro, Jamaica, NY, USA).

#### Spring-Mass Characteristics

From the assessments of t_c_, t_f_, running speed and from body mass and lower limb length, the spring-mass parameters (McMahon and Cheng, 1990) were calculated using the computation method proposed by Morin et al. (2005). The vertical stiffness (k_vert_ in N·m^−1^) was calculated as a ratio of the maximal reaction force (F_max_ in N) to downward displacement of the center of mass (Δy in m; Equation 1)

(1) $$\begin{array}{r} k_{vert}=\frac{F_{\max}}{\Delta y} \end{array}$$

with

(2) $$\begin{array}{r} F_{\max}=mg\;\frac{\pi }{2}\;\left(\frac{t_{f}}{t_{c}}+1\right) \end{array}$$

and

(3) $$\begin{array}{r} \Delta y=|-\frac{F_{\max}}{m}.\frac{{t_{c}}^{2}}{\pi^{2}}+g.\frac{{t_{c}}^{2}}{8}| \end{array}$$

where m is the runner's body mass (kg) and g is the acceleration due to gravity (100% BW: 1 g, 9.81 m·s^−2^; 60% BW: 0.6 g, 5.89 m·s^−2^; 20% BM: 0.2 g, 1.96 m·s^−2^), t_f_ is the fly time (s), and t_c_ is the contact time (s).

The leg stiffness (k_leg_ in N·m^−1^) was also calculated (Equation 4):

(4) $$\begin{array}{r} k_{leg}=\frac{F_{\max}}{\Delta L} \end{array}$$

where F_max_ is the maximal vertical ground reaction force during contact (N) and ΔL is the peak displacement of the leg spring (m) calculated from (Equation 5)

(5) $$\begin{array}{r} \Delta L=L-\sqrt{{L^{2}-\left(\frac{v.t_{c}}{2}\right)}^{2}}+\Delta y \end{array}$$

where L is the initial leg length (great trochanter to ground distance in a standing position) (m), v is the running velocity (m·s^−1^), t_c_ is the contact time (s) and Δy is the downward displacement of the center of mass (m; Equation 3).

Elastic energy (EL) storage during running (J; Equation 6) and natural frequency of the spring-mass system (Hz; Equation 7) was calculated:

(6) $$\begin{array}{r} EL=\frac{F_{max\;}\cdot \Delta L}{2} \end{array}$$

where F_max_ is the maximal ground reaction force during contact (N) and ΔL is the peak displacement of the leg spring (m). Then, EL value was divided by the step length and body mass to obtain EL per kg and unit distance (J·kg^−1^·m^−1^).

(7) $$\begin{array}{r} Natural\;step\;frequency=\frac{1}{2\pi }\cdot \sqrt{\frac{k_{vert}}{m}} \end{array}$$

where k_vert_ is the runner's vertical stiffness (N·m^−1^) and m is the runner's body mass (kg).

According to McMahon and Cheng (1990), we also calculated the angle of the lower limb spring at the initial ground contact relative to the vertical (θ; the half angle swept by the stance lower limb; Equation 8):

(8) $$\begin{array}{r} \theta =\sin^{-1}\left(\frac{v\cdot t_{c}}{2\cdot L}\right) \end{array}$$

where v is the running velocity (m·s^−1^), t_c_ contact time during support (s) and L is the initial leg length.

#### Statistical Analysis

All values are reported in the text as the mean ± SD. A t-test was used to compare the slope of the linear regression between $\overset{\cdot }{V}$O_2_ and time during the last minute of running for each speed with 0 (i.e., “perfect” steady state). The AIC (Akaike Information Criterion) system was used to determine the best modeling between the linear and curvilinear models:

(9) $$\begin{array}{r} AIC=N\times ln\left(RSS/N\right)+2K \end{array}$$

where N is the number of data points used in the analysis for each participant, RSS is the residual sum of squares from the linear or curvilinear model, and K is the number of parameters in the fitted model + 1 (3 for linear and 4 for curvilinear). The AIC was calculated for the linear and curvilinear models. The model with the lowest AIC was the more correct one and was confirmed by the difference (ΔAIC) between the 2 models:

(10) $$\begin{array}{r} \Delta AIC=N\times ln\left(RSS_{P}/RSS_{L}\right)+2\left({\text{K}}_{\text{P}}-{\text{K}}_{\text{L}}\right) \end{array}$$

A negative value of the ΔAIC suggests that curvilinear model, in the numerator, is better than linear model, whereas a positive value means that the linear model, in the denominator, is better than curvilinear model.

A two-way repeated measures ANOVA [gravity (20, 60, and 100% BW) × running speed (2.25, 3.17, 4.08, and 5.00 m·s^−1^)] was performed to compare energetics and biomechanics of running at each speed and gravity condition. The significance was determined with a t-test, with Bonferroni adjustment, when ANOVA revealed significant interaction effects. A one-way repeated measures non-parametric ANOVA of Friedman was used to compare the optimal running speeds determined from the speed-Cr relationships. At 100% BW, correlations between the difference between Cr at 5 m·s^−1^ and Cr at 4.08 m·s^−1^ (ΔCr_4−3_) and the difference between EL at 5 m·s^−1^ and EL at 4.08 m·s^−1^ (ΔEL_4−3_) and between the difference between Cr at 3.17 m·s^−1^ and Cr at 2.25 m·s^−1^ (ΔCr_2−1_) and the difference between EL at the same speeds (ΔEL_2−1_) were performed using the Pearson correlation coefficient (r). The level of significance was set at *P* ≤ 0.05.

## Results

### Energy Cost of Running

#### Steady State of the Oxygen Uptake

The slope of the linear regression between $\overset{\cdot }{V}$O_2_ and time during the last minute of running for each speed was not significantly different from 0 (data not shown; *P* ≥ 0.3) confirming that the steady state was achieved during the last minute of each speed by all runners.

#### Energy Cost Per kg of Body Mass

**Figure 1:**
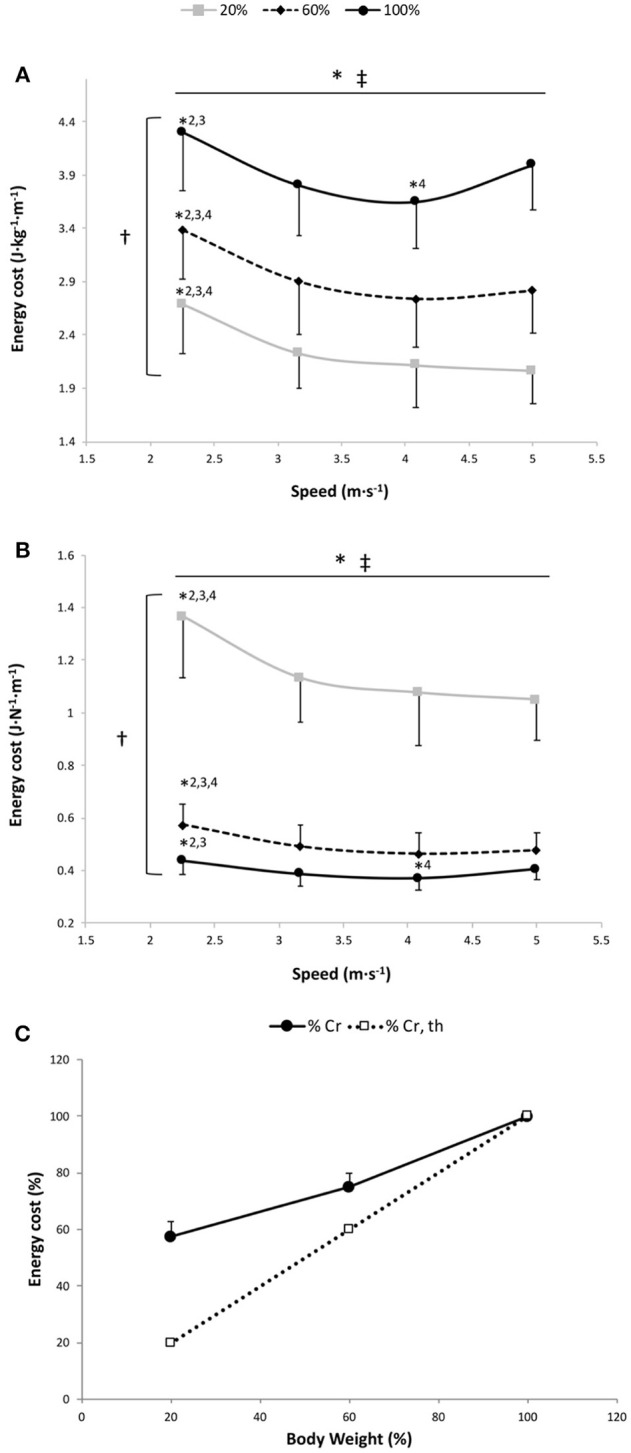
Energy cost per kg of body mass **(A)**, energy cost per body weight **(B)** and energy cost of running (Cr) as a percentage of 100% body weight **(C)** vs. running speed at 100% body weight (BW; 1 *g*), 60% BW (0.6 *g*), and 20% BW (0.2 *g*) (*n* = 12). Values are mean ± SD. ^*^P < 0.05 for the significant speed effect; ^†^*P* < 0.05 for the significant gravity effect; ^‡^*P* < 0.05 for the significant interaction effect; ^*^2 for significant difference from 3.17 m·s^−1^; ^*^3 for significant difference from 4.08 m·s^−1^; and ^*^4 for significant difference from 5.0 m·s^−1^ (*P* < 0.05). There was a significant gravity effect for each speed (*P* < 0.001; for sake of clarity, these significant differences are not shown). The dashed line in **(C)** represents a proportional decrease in Cr relative to BW (%Cr,th).

The two-way RM ANOVA revealed a main effect of gravity (*P* < 0.001); Cr was significantly different at all measured %BW (Figure 1A) but less than in direct proportion to BW (Figure 1C). A significant main speed effect (*P* < 0.001) was found, suggesting that Cr changed with running speeds for the 3 gravity conditions (Figure 1A). This statistically confirmed the AIC system results attesting that the curvilinear modeling better fit the speed-Cr relationship than the linear model (see below). The speed × gravity interaction effect was also significant (*P* = 0.014) (Figure 1A). In fact, Cr was significantly higher at 2.25 m·s^−1^ than at the other speeds for 20 and 60% BW conditions (*P* ≤ 0.001). Whereas at 100% BW, Cr was significantly greater at 2.25 m·s^−1^ than at 3.17 and 4.08 m·s^−1^ (*P* = 0.001) and was significantly lower at 4.08 m·s^−1^ than at 5.00 m·s^−1^ (*P* = 0.031; Figure 1A).

#### Energy Cost Per Body Weight

The two-way RM ANOVA revealed a main effect of gravity (*P* < 0.001); Cr normalized to the gravity was significantly different at all measured %BW (Figure 1B). Significant main speed and speed × gravity interaction effects were also found (*P* < 0.001; Figure 1B). For 60 and 20% BW conditions, Cr was significantly higher at 2.25 m·s^−1^ than at the other speeds (*P* ≤ 0.001). While at 100% BW, Cr was significantly higher at 2.25 m·s^−1^ than at 3.17 and 4.08 m·s^−1^ (*P* = 0.001) and was significantly lower at 4.08 m·s^−1^ than at 5.00 m·s^−1^ (*P* = 0.031).

#### Linear and Curvilinear Models for the Speed-Cr Per kg of Body Mass Relationship and Cr Optimal Running Speeds

For the speed-Cr relationship, 11 out of 12 runners had a negative ΔAIC, suggesting that the curvilinear model provided a better fit than the linear model for the 3 conditions (ΔAIC equation: −5.9 ± 5.6, −7.0 ± 7.0 and −8.7 ± 7.0 at 20, 60, and 100% BW, respectively). The mean *r*^2^ for fitting a curvilinear model to the speed-Cr relationship across all participants was 0.93 ± 0.08, 0.91 ± 0.12, and 0.87 ± 0.18 at 20, 60, and 100% BW, respectively, while the mean *r*^*2*^ with the linear model was only 0.65 ± 0.28, 0.59 ± 0.24, and 0.29 ± 0.28 at 20, 60, and 100% BW, respectively. There were no significant differences in Cr running optimal speeds among the 3 gravity conditions (100% BW: 3.90 ± 0.31 m·s^−1^; 60% BW: 4.21 ± 0.24 m·s^−1^; and 20% BW: 4.18 ± 1.23 m·s^−1^; *P* = 0.54).

### Spatiotemporal Parameters

#### Contact Time

**Figure 2:**
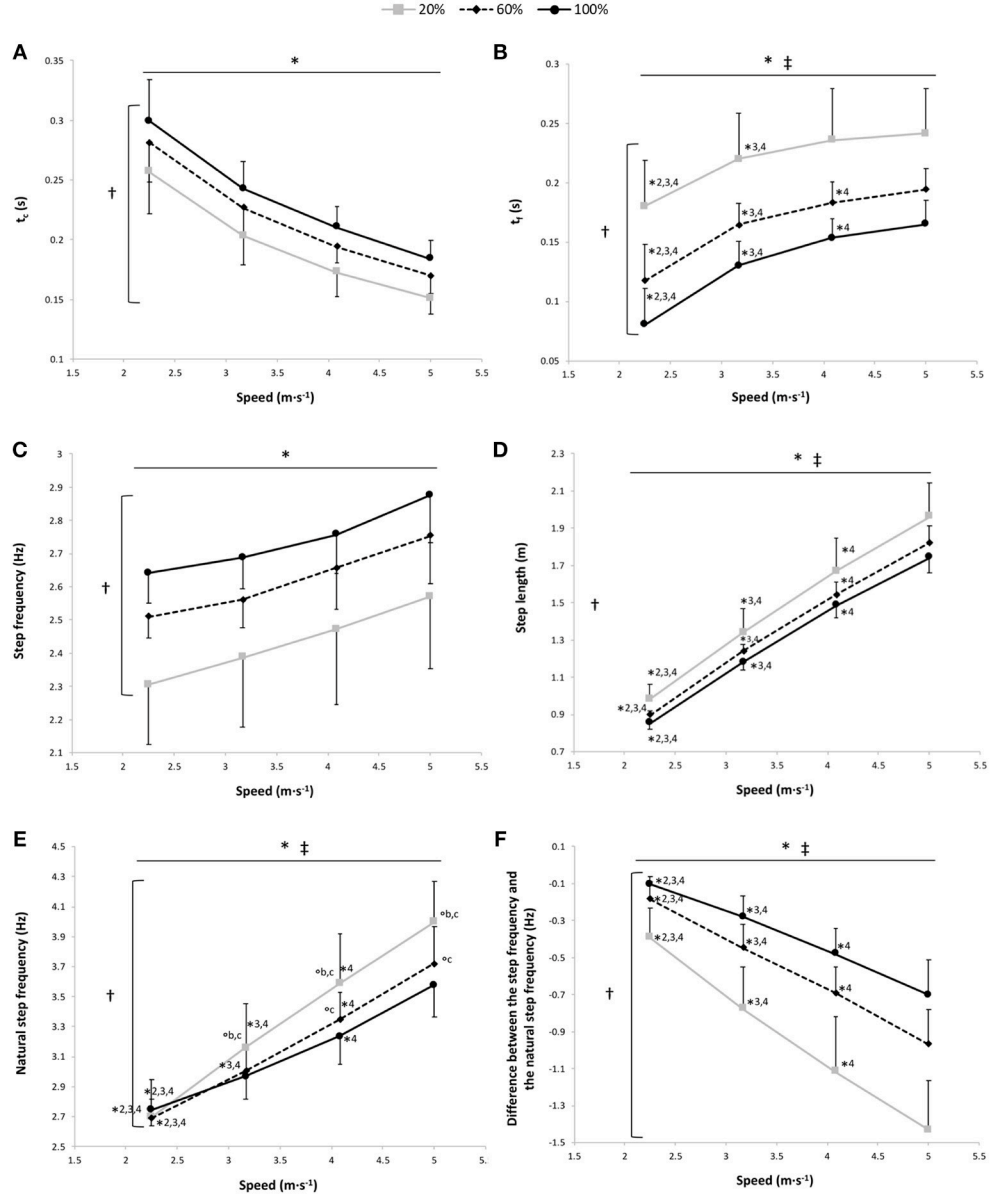
Contact time (t_c_) **(A)**, flight time (t_f_) **(B)**, step frequency **(C)**, step length **(D)**, natural step frequency **(E)**, and the difference between the step frequency and the natural step frequency **(F)** vs. running speed at 100% body weight (BW; 1 *g*), 60% BW (0.6 *g*) and 20% BW (0.2 *g*) (n = 12). Values are mean ± SD. ^*^P < 0.05 for the significant speed effect; ^†^*P* < 0.05 for the significant gravity effect; ^‡^*P* < 0.05 for the significant interaction effect; ^*^2 for significant difference from 3.17 m·s^−1^; ^*^3 for significant difference from 4.08 m·s^−1^, ^*^4 for significant difference from 5.0 m·s^−1^ (*P* < 0.05); ^°a^ for significant difference from 20% BW; ^°b^ for significant difference from 60% BW; and ^°c^ for significant difference from 100% BW (*P* < 0.05). For the graphs b, d, and f, there was a significant gravity effect for each speed (*P* < 0.001; for sake of clarity, these significant differences are not shown).

A significant main gravity effect (*P* < 0.001) was found, t_c_ was significantly different at all measured %BW, with a significant decrease in t_c_ depending on the speed for the 3 gravity conditions (*P* < 0.001) and with no speed x gravity interaction effect (*P* = 0.44; Figure 2A).

#### Flight Time

The two-way RM ANOVA revealed a main effect of gravity (*P* < 0.001); t_f_ was significantly different at all measured %BW, with a significant main speed effect showing that t_f_ increased with running speeds for the 3 gravity conditions (*P* < 0.001; Figure 2B). There was a significant speed × gravity interaction effect (*P* = 0.001; Figure 2B). At 100, 60, and 20% BW, t_f_ was significantly lower at 2.25 and 3.17 m·s^−1^ than at the other speeds (*P* ≤ 0.01). Whereas only at 100% and at 60% BW was t_f_ significantly lower at 4.08 m·s^−1^ than at 5.00 m·s^−1^ (*P* = 0.037 and *P* < 0.001, respectively).

#### Step Frequency

The step frequency was significantly higher at 100% BW than at 60% and 20% BW and at 60% BW than at 20% BW (main gravity effect: *P* < 0.001; Figure 2C). There was a significant speed effect showing a significant increase of the step frequency depending on speed for the 3 gravity conditions (*P* < 0.001; Figure 2C), with no speed × gravity interaction effect (*P* = 0.61).

#### Step Length

A significant main gravity effect (*P* = 0.001) was found, the step length was significantly different at all measured %BW (*P* ≤ 0.016), with a significant main speed effect showing an increase in the step length with running speeds for the 3 gravity conditions (*P* < 0.001; Figure 2D).

### Spring-Mass Characteristics

#### Maximal Vertical Ground Reaction Force

**Figure 3:**
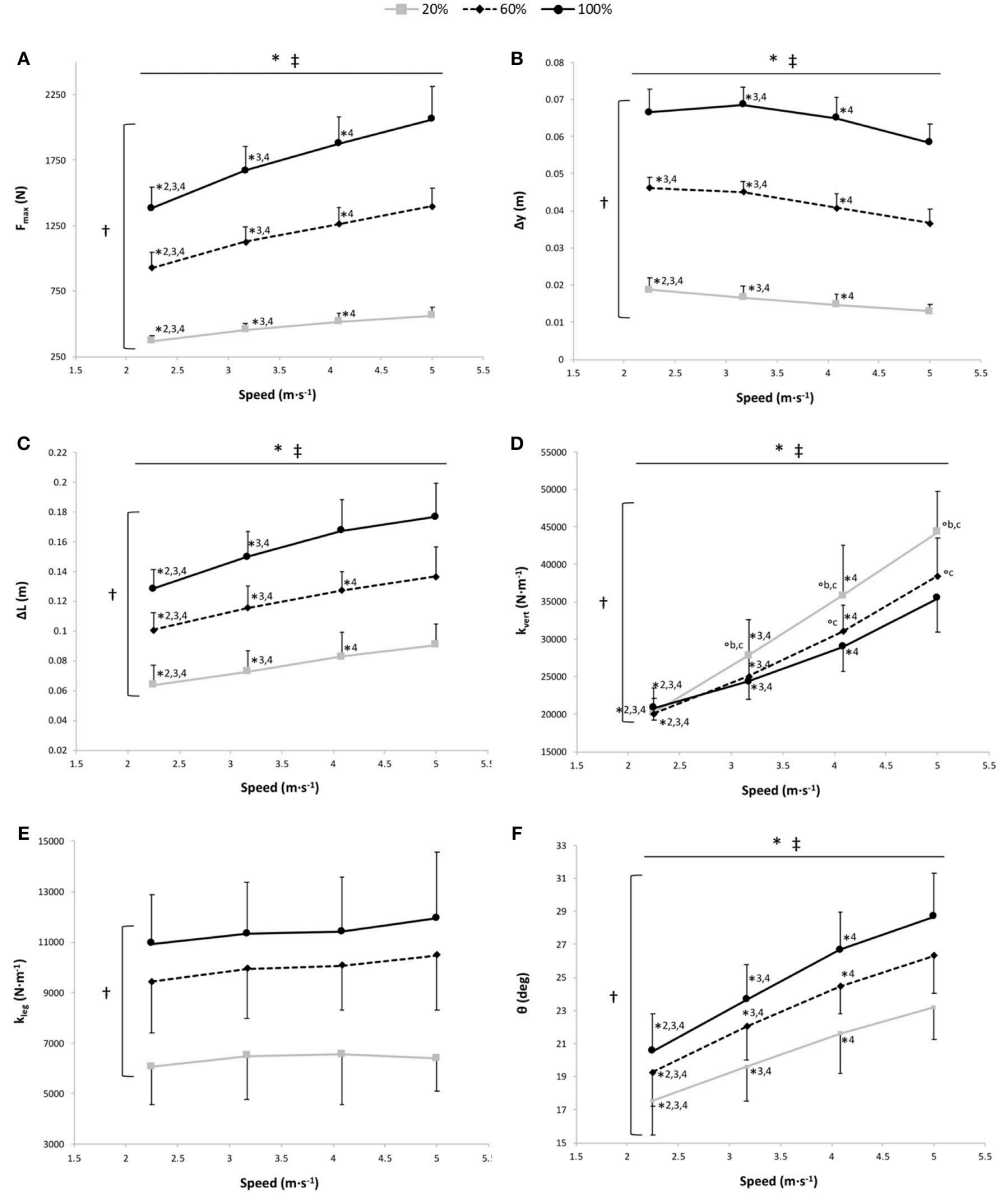
Maximal vertical ground reaction force (F_max_) **(A)**, vertical displacement of the center of mass (Δy) **(B)**, lower limb length variation (compression) during contact (ΔL) **(C)**, vertical stiffness (k_vert_) **(D)**, leg stiffness (k_leg_) **(E)**, and θ angle **(F)** vs. running speed at 100% body weight (BW; 1 *g*), 60% BW (0.6 *g*), and 20% BW (0.2 *g*) (*n* = 12). Values are mean ± SD. ^*^P < 0.05 for the significant speed effect; ^†^*P* < 0.05 for the significant gravity effect; ^‡^*P* < 0.05 for the significant interaction effect; ^*^2 for significant difference from 3.17 m·s^−1^; ^*^3 for significant difference from 4.08 m·s^−1^, ^*^4 for significant difference from 5.00 m·s^−1^ (*P* < 0.05); ^°a^ for significant difference from 20% BW; ^°b^ for significant difference from 60% BW; and ^°c^ for significant difference from 100% BW (*P* < 0.05). For the graphs a, b, c, and f, there was a significant gravity effect for each speed (*P* < 0.001; for sake of clarity, these significant differences are not shown).

The two-way RM ANOVA revealed a main effect of gravity (*P* < 0.001); F_max_ was significantly different at all measured %BW, with a significant increase in F_max_ depending on speed for the 3 gravity conditions (*P* < 0.001; Figure 3A).

#### Vertical Displacement of the Center of Mass

A significant main gravity effect (*P* < 0.001) was found, Δy was significantly different at all measured %BW (*P* < 0.001), with a significant main speed effect showing a decrease in the Δy with running speeds for the 3 gravity conditions (*P* < 0.001; Figure 3B). The speed x gravity interaction effect was also significant (*P* = 0.013; Figure 3B). At 20% BW, Δy was significantly greater at 2.25 m·s^−1^ than at the other speeds (*P* < 0.001). At 60% BW, Δy was significantly higher at 2.25 m·s^−1^ than at 4.08 and 5.00 m·s^−1^ (*P* = 0.008 and *P* < 0.001, respectively). For all gravity conditions, Δy was significantly higher at 3.17 m·s^−1^ than at 4.08 m·s^−1^ (*P* ≤ 0.006) and 5.00 m·s^−1^ (*P* < 0.001) and at 4.08 m·s^−1^ than at 5.00 m·s^−1^ (*P* ≤ 0.001).

#### Lower Limb Length Variation (Compression) During Contact

The two-way RM ANOVA revealed a main effect of gravity (*P* < 0.001); ΔL was significantly different at all measured %BW, with a significant main speed effect showing an increased ΔL with running speeds for the 3 gravity conditions (*P* < 0.001; Figure 3C). A significant speed × gravity interaction effect was also found (*P* < 0.001; Figure 3C). For each gravity condition, ΔL was significantly lower at 2.25 m·s^−1^ than at the other speeds (*P* ≤ 0.013) and at 3.17 m·s^−1^ than at 4.08 and 5.00 m·s^−1^ (*P* ≤ 0.001). However, only at 60 and 20% BW was ΔL significantly lower at 4.08 m·s^−1^ than at 5.00 m·s^−1^ (*P* ≤ 0.022).

#### Vertical Stiffness

The two-way RM ANOVA revealed a main effect of gravity (*P* < 0.001); k_vert_ was significantly different at all measured %BW, with significant main speed and speed × gravity interaction effects (*P* < 0.001 for both; Figure 3D). k_vert_ increased with the running speed for the 3 gravity conditions. Within the speeds, the gravity effect was significant at 4.08 m·s^−1^ (*P* ≤ 0.009) and at 5.00 m·s^−1^ (*P* ≤ 0.001). Whereas at 3.17 m·s^−1^, k_vert_ was significantly greater at 20% BW than at the 2 other gravity conditions (*P* ≤ 0.038), and at 2.25 m·s^−1^, there was no significant difference in k_vert_ among the 3 gravity conditions (*P* = 1 and *P* = 0.055).

#### Leg Stiffness

A significant main gravity effect (*P* < 0.001) was found, k_leg_ was significantly different at all measured %BW (*P* < 0.001), with no significant main speed and speed × gravity interaction effects (*P* = 0.08 and *P* = 0.38, respectively; Figure 3E).

#### Elastic Energy Storage

**Figure 4:**
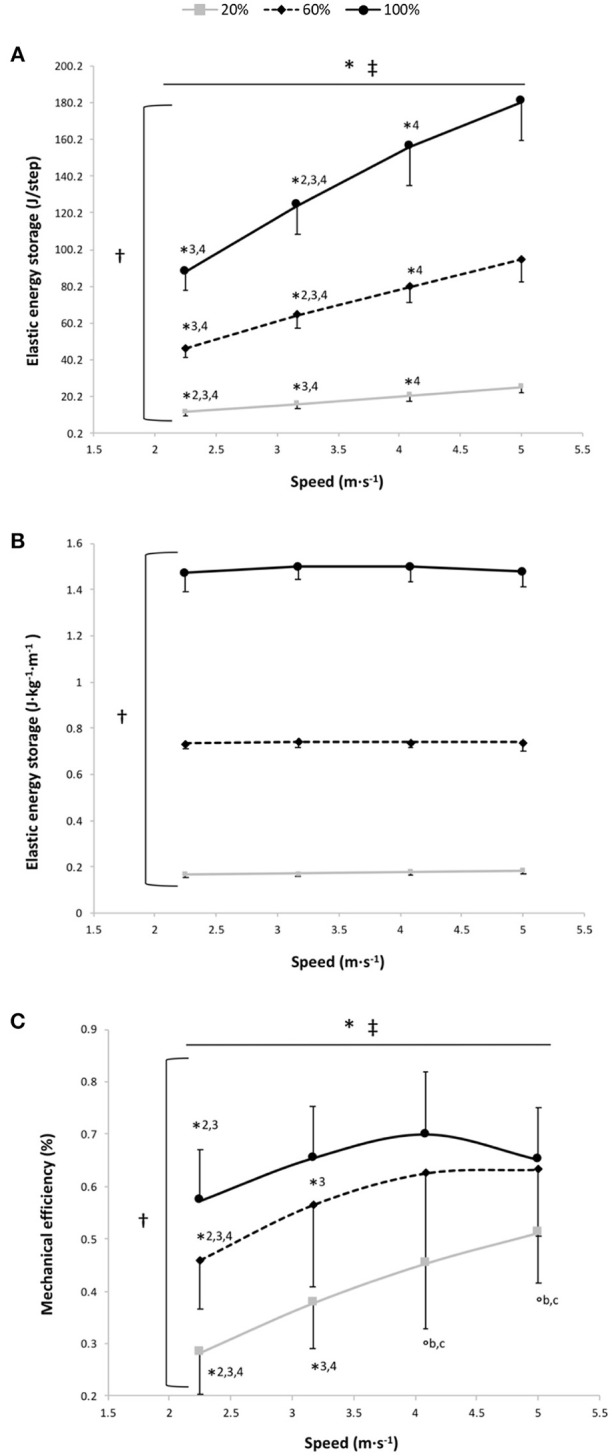
Elastic energy storage (EL) per step **(A)**, EL per kg of body mass and unit distance **(B)** and mechanical efficiency **(C)** vs. running speed at 100% body weight (BW; 1 *g*), 60% BW (0.6 *g*) and 20% BW (0.2 *g*) (*n* = 12). Values are mean ± SD. ^*^P < 0.05 for the significant speed effect; ^†^*P* < 0.05 for the significant gravity effect; ^‡^*P* < 0.05 for the significant interaction effect; ^*^2 for significant difference from 3.17 m·s^−1^; ^*^3 for significant difference from 4.08 m·s^−1^, ^*^4 for significant difference from 5.0 m·s^−1^ (*P* < 0.05); ^°b^ for significant difference from 60% BW; and ^°c^ for significant difference from 100% BW (*P* < 0.05). For the graphs a and b, there was a significant gravity effect for each speed (*P* < 0.001; for sake of clarity, these significant differences are not shown).

EL, expressed in J·step^−1^, was significantly higher at 100% BW than at 60 and 20% BW and at 60% BW than at 20% BW (main gravity effect: *P* < 0.001) with a significant increase in EL per step depending on speed for the 3 gravity conditions (*P* < 0.001; Figure 4A). For EL expressed in J·kg^−1^·m^−1^, the two-way RM ANOVA revealed a main effect of gravity (*P* < 0.001); EL storage per unit distance was significantly different at all measured %BW (*P* < 0.001), with no significant main speed and speed × gravity interaction effects (*P* = 0.39 and *P* = 0.37, respectively; Figure 4B). At 100% BW, ΔCr_4−3_ tended to be negatively correlated to ΔEL_4−3_ (*r* = 0.55; *P* = 0.067), whereas there was no significant correlation between ΔCr_2−1_ and ΔEL_2−1_ (*r* = 0.41; *P* = 0.18).

#### Natural Step Frequency

A significant main gravity effect (*P* < 0.001) was found, the natural step frequency was significantly different at all measured %BW (*P* < 0.001), with significant main speed and speed × gravity interaction effects (*P* < 0.001 for both; Figure 2E). The natural step frequency increased with running speeds. Within the speeds, the gravity effect was significant at 4.08 m·s^−1^ (*P* ≤ 0.007) and at 5.00 m·s^−1^ (*P* ≤ 0.001). Conversely, at 3.17 m·s^−1^, the natural step frequency was significantly higher at 20% BW than at the 2 other gravity conditions (*P* ≤ 0.043), and at 2.25 m·s^−1^, there was no significant difference in the natural step frequency among the 3 gravity conditions (*P* ≥ 0.054).

#### The Difference Between the Step Frequency and the Natural Step Frequency

The difference between the step frequency adopted and the natural step frequency was significantly lower at 100% BW than at 60 and 20% BW and at 60% BW than at 20% BW (main gravity effect: *P* < 0.001; Figure 2F). A significant main speed effect was also found with a significant increase in this difference depending on speed for the 3 gravity conditions (*P* < 0.001; Figure 2F).

#### Angle of the Lower Limb at the Initial Ground Contact Relative to the Vertical

The two-way RM ANOVA revealed a main effect of gravity (*P* < 0.001); θ was significantly different at all measured %BW, with a significant main speed effect showing an increase in θ depending on speed for the 3 gravity conditions (*P* < 0.001; Figure 3F).

## Discussion

The main findings of the present study were that, under normal gravity conditions (100% BW), (1) the speed-Cr relationship was curvilinear; (2) the elastic energy per step increased with speed; and (3) the elastic energy per unit distance was independent of the running speed. Partially in contrast with our hypothesis, EL/distance was not reduced at the slowest and fastest running speeds but was maintained constant at these speeds. This may occur only at the expense of increased muscle activation due to a more critical control of musculotendon length at slow running speeds (Seyfarth et al., 2002; Sasaki and Neptune, 2006), or to counter the shift toward a non-optimal operating regions of muscle fibers on their force-length curves at fast running speeds (Lai et al., 2014), both inducing an increase in Cr at these speeds. This U-shaped speed-Cr per kg of body mass relationship was also found in reduced gravity (20 and 60% BW) and was downward shifted compared to 100% BW, attesting to the pivotal role of the body weight transported on Cr (Kram and Taylor, 1990). However, these reduced gravity conditions were characterized by a reduced contribution of SSC with decreased EL and k_leg_ compared to that under normal terrestrial gravitational conditions, leading to a greater Cr normalized by BW actually transported (i.e., higher cost of force generation). These mechanisms may contribute to the less than proportional decrease in metabolic cost per kg of body mass of running as a function of reduced gravity (Teunissen et al., 2007; Grabowski et al., 2010; Raffalt et al., 2013).

At 100% BW, our findings showed a significant speed effect with a higher Cr at 2.25 (+15% compared to intermediate speeds) and 5.00 m·s^−1^ (+7% compared to intermediate speeds) and a plateau at intermediate speeds (3.17 and 4.08 m·s^−1^). Moreover, negative ΔAIC values (−8.7 ± 7.0) and higher *r*^2^ values for the curvilinear model (0.87 ± 0.18) compared with those obtained for the linear model (0.29 ± 0.28) were found demonstrating a U-shaped relationship between Cr and speed, corroborating recent findings (Steudel-Numbers et al., 2007; Fletcher et al., 2009; Steudel-Numbers and Wall-Scheffler, 2009; Willcockson and Wall-Scheffler, 2012; Shaw et al., 2013; Rathkey and Wall-Scheffler, 2017; Black et al., 2018) and in contrast with the “classic” body of evidence (Hagan et al., 1980; Kram and Taylor, 1990; Bramble and Lieberman, 2004). This highlights that running, similar to walking (Saibene and Minetti, 2003), has an optimal economy at intermediate speeds (~3.5–4.4 m·s^−1^), which confirms existence of an optimal speed.

Although EL per kg and unit distance was independent of the speed, our findings showed that at the lowest and fastest speeds the EL/distance was slightly but no significantly reduced (−1.5%) and ΔCr_4−3_ tended to be negatively correlated to ΔEL_4−3_. This partially confirms our hypothesis that the storage/release of EL per unit distance may at least contribute to increase the Cr at the extremities of the speed-Cr relationship, especially at fast running speed. In fact, even though EL/step increased with running speeds, the concomitant increase in step length makes the speed-EL/distance relationship relatively flat and penalizes the storage and return of EL at fast running speeds. We can speculate that this maintained or slightly reduced EL per unit distance at these speeds occurs only through a greater volume of active muscle recruited to counter the increasing the less favorable contractile conditions during fiber contractions (Lai et al., 2014; Kipp et al., 2018). In fact, Lai et al. (2014) previously showed that, with increasing running speeds, the muscle fibers operate on the ascending and unfavorable part of the force-length relationship with a concomitant increase in electromyography activity reflecting a greater muscle activation. This results in an increased Cr at these speeds (Lai et al., 2014; Kipp et al., 2018). At slow running speeds, the EL per unit distance may also be maintained (or slightly reduced) only increasing the neuromotor control of the muscle fibers recruited because the lower limb adjustment becomes more critical to preserve a stable bouncing gait (Seyfarth et al., 2002; Sasaki and Neptune, 2006). This increased neuromotor control of the fibers may also induce an increased Cr at these speeds (Biewener and Roberts, 2000; Sasaki and Neptune, 2006).

The findings of the present study do not corroborate the recent results of Fletcher and MacIntosh (Fletcher and MacIntosh, 2015) for which the amount of tendon strain energy released represents a small portion of the Cr and that for reducing this latter is more important to decrease the muscle energy cost through a reduction in muscle fascicle shortening during running (i.e., less energy release from the tendon). However, these findings result from a series of indirect estimates assessed only at the ankle level. At 3.17 m·s^−1^, our findings showed that 1.49 J·kg^−1^·m^−1^ of EL was stored, which corresponds to the external mechanical work (1.30 J·kg^−1^·m^−1^: mean value at similar speed for a group of similar running expertise and performance; unpublished data) or in line with previous results (Willems et al., 1995). This corroborates that the leg spring behaves as a simple linear spring because the work performed by the spring is similar to the mechanical work of the center of mass during running (i.e., the external mechanical work; Farley et al., 1993). For each runner and for all speeds, we estimated the total mechanical work as the sum of the external mechanical work, estimated by using the work performed by the spring (Farley et al., 1993), and the internal mechanical work assessed using the formula of Nardello et al. (2011). Then, we divided this estimate of the total mechanical work by Cr to obtain an estimate of the mechanical efficiency (i.e., the overall efficiency of locomotor apparatus taking into account the work necessary to maintain motion and the chemical energy transformed by the muscles) (Cavagna and Kaneko, 1977) (Figure 4C). Our values are in line with those previously reported but in contrast to the linear increase of the mechanical efficiency with speed (Cavagna and Kaneko, 1977; Willems et al., 1995). In fact, at 100% BW, our data show that the speed-mechanical efficiency relationship is an inverted U-shaped relationship with a significant main speed effect (*P* < 0.001). Mechanical efficiency was significantly lower at 2.25 m·s^−1^ than at 3.17 and 4.08 m·s^−1^ (*P* ≤ 0.002) and its value at 5 m·s^−1^ decreased (no significantly) compared to that at 4.08 m·s^−1^ (*P* = 0.56; Figure 4C). As for Cr, the mechanical efficiency is penalized at the extremities of the curve because the runners may maintain EL/distance at these speeds only at the expense of increased muscle activation. Moreover, at this gravity condition, the speed-mechanical efficiency relationship was curvilinear for all runners (*r*^2^ = 0.87 ± 0.17) and the mechanical efficiency optimal running speed (3.65 ± 0.90) was similar to the Cr optimal running speed (3.90 ± 0.31; *P* = 0.34). This confirms that the “trade-offs between control and efficiency” (Sasaki and Neptune, 2006) may influence the choice of the optimal running speed as previously reported, using forward dynamic simulations, for the preferred gait walk-run transition speed (Sasaki and Neptune, 2006). Moreover, our optimal speed (3.9 m·s^−1^) was close to that previously found (~3.5 m·s^−1^) by others (Steudel-Numbers et al., 2007; Steudel-Numbers and Wall-Scheffler, 2009; Willcockson and Wall-Scheffler, 2012; Rathkey and Wall-Scheffler, 2017; Black et al., 2018). This Cr optimal running speed may be useful to distance running performance, especially in ultrarunning (Rathkey and Wall-Scheffler, 2017).

In addition, other mechanical determinants may be involved in the increased Cr at faster running speeds and thus in the U-shaped speed-Cr relationship. First, at these speeds, the greater joint angular velocities and reduction of t_c_, as shown in our findings, require greater rates of force development associated with less efficient recruitment of type II motor units during the contact phase (Shaw et al., 2014; Fletcher and MacIntosh, 2017). This is in line with the “cost of generating force hypothesis,” suggesting that the metabolic rate is proportional to BW and inversely proportional to the time of foot-ground contact during running (Kram and Taylor, 1990). However, others (Minetti et al., 1994; Nummela et al., 2007) did not confirm this hypothesis at fast running speeds showing significant negative correlations between Cr and t${}_{\text{c}}^{-1}$. Therefore, the relationship between Cr and t_c_ is not yet clearly resolved and should be further investigated (see the recent reviews Lacour and Bourdin, 2015; Folland et al., 2017) for more details. Second, our findings showed that t_f_ and step frequency increase with running speed, corroborating previous results (Donelan and Kram, 2000; Grabowski and Kram, 2008; Raffalt et al., 2013). Above 3.6 m·s^−1^, it has been suggested (Cavagna et al., 1988; Lacour and Bourdin, 2015) that effective flight time becomes progressively longer than effective contact time, and the step frequency becomes gradually lower than the natural step frequency (Figure 2) inducing an asymmetric rebound, an alteration of the spring-mass system and an increased mechanical energy to maintain the running oscillations attested by the increased k_vert_ with speed (Figure 3D). These changing mechanics of running at fast speed may contribute to the increased Cr at 5.00 m·s^−1^ reported in the present study. However, the relative rate in k_vert_ increasing seems to be relatively constant as a function of the speed, and thus, these mechanical changes may not be the main factors to explain the decreased running economy at 5.00 m·s^−1^.

The U-shaped speed-Cr relationship was also shown in reduced gravity conditions (20% and 60% BW) with a significant speed effect for Cr, negative ΔAIC values (20% BW: −5.9 ± 5.6 and 60% BW: −7.0 ± 7.0) and higher r^2^ values for the curvilinear model (20% BW: 0.93 ± 0.08 and 60% BW: 0.91 ± 0.12) than those obtained for the linear model (20% BW: 0.65 ± 0.28 and 60% BW: 0.59 ± 0.24). However, differently from 100% BW, Cr was higher only at the slowest speed (2.25 m·s^−1^) compared to the other speeds (3.17, 4.08, and 5.00 m·s^−1^) (Figure 1A). In both conditions, a faster running speed would have been necessary to increase Cr as demonstrated at 100% BW for the fastest speeds. The greater Cr at 2.25 m·s^−1^ compared to other speeds may be explained by the same mechanical mechanism, which occurs at 100% BW and is likely related to maintaining EL/distance stored and released in the muscle-tendon unit only thanks to an increased muscle activation at the slowest speed. As hypothesized, our findings showed a downward shift of the speed-Cr per kg of body mass relationship with gravity (Figure 1A) but less than that in direct proportion to BW (20% BW: −42 and 60% BW: −25%; Figure 1C). This corroborates the crucial role of BW in Cr previously reported (i.e., the “cost of generating force hypothesis” of Kram and Taylor, 1990) and this less than proportional decrease in the metabolic cost of running relative to BW (Teunissen et al., 2007; Grabowski and Kram, 2008; Lacour and Bourdin, 2015) suggests that the latter is not the only factor involved in decreasing Cr. The decreased t_c_ for the same running speed in reduced gravity conditions, reported in the present study and by others (He et al., 1991), may explain the less than proportional decrease in Cr with respect to the reduction in the transported BW under reduced gravity. In fact, a reduced t_c_ would necessitate a recruitment of less efficient higher threshold motor units inducing an increase of Cr (Shaw et al., 2014; Fletcher and MacIntosh, 2017). Normalizing Cr by BW (J·N^−1^·m^−1^) allowed us to study the effect of the gravity on Cr independent of change of BW. These results showed a significant gravity factor for the speed-Cr relationship, with a higher Cr at 20% BW than at 60 and 100% BW and at 60% than at 100% BW (the opposite of the relationship of Cr-speed expressed per kg of transported mass). This increase in the Cr (J·N^−1^·m^−1^) with the reduced gravity is likely due to a reduced contribution of SSC associated with a significant decrease in k_leg_ (Donelan and Kram, 2000) and EL and a significant increase in k_vert_ (He et al., 1991; Sainton et al., 2015). Consequently, more mechanical energy must be actively “injected” by the muscle-tendon unit in the system, which may contribute to the increased Cr with the reduced gravity conditions (i.e., higher cost of force generation).

Some methodological limitations exist and need to be addressed. First, the findings of this study are obtained using a simple and valid computational method (based on a sine-wave modeling force time curves; Morin et al., 2005) that estimates the parameters of the spring-mass model from few simple anthropometric and mechanical parameters. These methods include many assumptions and limitations (Blickhan, 1989; McMahon and Cheng, 1990; He et al., 1991; Farley and González, 1996; Morin et al., 2005) that may restrict our conclusion on the underlying mechanisms. However, due to the methodological challenges associated to directly measure all relevant parameters *in vivo* and under dynamic conditions in order to understand the energetics and the role of muscle-tendon unit during running, the use of spring-loaded inverted pendulum model seems rational and relevant. Second, Grabowski and Kram (2008) showed that, due to the interface between the chamber and runner, the AlterG® device applied a forward directed force to the runner in reduced gravity conditions. This altered the braking phase and decreased Cr. However, contrary to this previous study and for minimizing this methodological problem, our participants also run in the AlterG® device during 100% BW condition and we advised them to run in the middle of the chamber aperture to minimize the horizontal assistance (Grabowski and Kram, 2008). Third, the same authors (Grabowski and Kram, 2008) showed that, at 25% BW, the impact peak magnitude of the vertical ground reaction force was greater than the active peak of the vertical ground reaction force. This could be a limitation in using the computational method of Morin et al. (2005) to estimate the parameters of the spring-mass model at 20% BW. However, our values of the biomechanical parameters at 20% BW are in line with those of previous studies using a direct assessment of the ground reaction forces during treadmill running at similar gravity conditions (He et al., 1991; Donelan and Kram, 2000).

In conclusion, our findings showed that for the 3 gravity conditions, the speed-Cr relationship was curvilinear and the optimization of SSC and muscle activation in the muscle-tendon unit may be involved to explain these U-shaped relationships, especially at normal terrestrial gravitational conditions (100% BW). The U-shaped speed-Cr per kg of body mass relationship was shifted downward with decreased gravity, attesting to the pivotal role of the body weight transported on Cr. However, this decreased Cr per kg of body mass was disproportional to BW and related to a reduced contribution of SSC with decreased EL and k_leg_ in hypogravity compared with normal terrestrial gravitational conditions. Nevertheless, the optimal Cr running speed was similar under the 3 gravity conditions and therefore was independent of gravity. Future longitudinal studies should investigate the practical use of this speed in training intensity individualization in ultrarunning races.

## Author Contributions

AC, EF, and DM conceived and designed the experiments, performed the experiments, analyzed the data and interpreted results of research, wrote the paper, and were involved in the editing process of the manuscript.

### Conflict of Interest Statement

The authors declare that the research was conducted in the absence of any commercial or financial relationships that could be construed as a potential conflict of interest.

## References

[B1] Astrand P. O. Rodahl K. (1986). Textbook of Work Physiology. New York, NY: McGraw-Hill Series in Health Ed.

[B2] Bassett D. R. Jr. Howley E. T. (2000). Limiting factors for maximum oxygen uptake and determinants of endurance performance. Med. Sci. Sports Exerc. 32, 70–84. 10.1097/00005768-200001000-00012 10647532

[B3] Biewener A. A. Roberts T. J. (2000). Muscle and tendon contributions to force, work, and elastic energy savings: a comparative perspective. Exerc. Sport Sci. Rev. 28, 99–107. 10916700

[B4] Black M. I. Handsaker J. C. Allen S. J. Forrester S. E. Folland J. P. (2018). Is there an optimal speed for economical running? Int. J. Sports Physiol. Perform. 13, 75–81. 10.1123/ijspp.2017-0015 28459289

[B5] Blickhan R. (1989). The spring-mass model for running and hopping. J. Biomech. 22, 1217–1227. 10.1016/0021-9290(89)90224-8 2625422

[B6] Bramble D. M. Lieberman D. E. (2004). Endurance running and the evolution of Homo. Nature 432, 345–352. 10.1038/nature03052 15549097

[B7] Cavagna G. A. Franzetti P. Heglund N. C. Willems P. (1988). The determinants of the step frequency in running, trotting and hopping in man and other vertebrates. J. Physiol. 399, 81–92. 10.1113/jphysiol.1988.sp017069 3404473 PMC1191653

[B8] Cavagna G. A. Kaneko M. (1977). Mechanical work and efficiency in level walking and running. J. Physiol. 268, 467–481. 10.1113/jphysiol.1977.sp011866 874922 PMC1283673

[B9] Cavagna G. A. Saibene F. P. Margaria R. (1964). Mechanical Work in Running. J. Appl. Physiol. 19, 249–256. 10.1152/jappl.1964.19.2.249 14155290

[B10] Donelan J. M. Kram R. (2000). Exploring dynamic similarity in human running using simulated reduced gravity. J. Exp. Biol. 203(Pt 16), 2405–2415. 10903155 10.1242/jeb.203.16.2405

[B11] Farley C. T. Ferris D. P. (1998). Biomechanics of walking and running: center of mass movements to muscle action. Exerc. Sport Sci. Rev. 26, 253–285. 10.1249/00003677-199800260-00012 9696992

[B12] Farley C. T. Glasheen J. McMahon T. A. (1993). Running springs: speed and animal size. J. Exp. Biol. 185, 71–86. 8294853 10.1242/jeb.185.1.71

[B13] Farley C. T. González O. (1996). Leg stiffness and stride frequency in human running. J. Biomech. 29, 181–186. 10.1016/0021-9290(95)00029-1 8849811

[B14] Fletcher J. R. Esau S. P. Macintosh B. R. (2009). Economy of running: beyond the measurement of oxygen uptake. J. Appl. Physiol. 107, 1918–1922. 10.1152/japplphysiol.00307.2009 19833811

[B15] Fletcher J. R. MacIntosh B. R. (2015). Achilles tendon strain energy in distance running: consider the muscle energy cost. J. Appl. Physiol. 118, 193–199. 10.1152/japplphysiol.00732.2014 25593218 PMC4297774

[B16] Fletcher J. R. MacIntosh B. R. (2017). Running economy from a muscle energetics perspective. Front. Physiol. 8:433. 10.3389/fphys.2017.00433 28690549 PMC5479897

[B17] Folland J. P. Allen S. J. Black M. I. Handsaker J. C. Forrester S. E. (2017). Running technique is an important component of running economy and performance. Med. Sci. Sports Exerc. 49, 1412–1423. 10.1249/MSS.0000000000001245 28263283 PMC5473370

[B18] Foster C. Lucia A. (2007). Running economy : the forgotten factor in elite performance. Sports Med. 37, 316–319. 10.2165/00007256-200737040-00011 17465597

[B19] Gojanovic B. Cutti P. Shultz R. Matheson G. O. (2012). Maximal physiological parameters during partial body-weight support treadmill testing. Med. Sci. Sports Exerc. 44, 1935–1941. 10.1249/MSS.0b013e31825a5d1f 22543742

[B20] Grabowski A. M. Kram R. (2008). Effects of velocity and weight support on ground reaction forces and metabolic power during running. J. Appl. Biomech. 24, 288–297. 10.1123/jab.24.3.288 18843159

[B21] Grabowski A. M. McGowan C. P. McDermott W. J. Beale M. T. Kram R. Herr H. M. (2010). Running-specific prostheses limit ground-force during sprinting. Biol. Lett. 6, 201–204. 10.1098/rsbl.2009.0729 19889694 PMC2865064

[B22] Hagan R. D. Strathman T. Strathman L. Gettman L. R. (1980). Oxygen uptake and energy expenditure during horizontal treadmill running. J. Appl. Physiol. Respir. Environ. Exerc. Physiol. 49, 571–575. 10.1152/jappl.1980.49.4.571 7440273

[B23] He J. P. Kram R. McMahon T. A. (1991). Mechanics of running under simulated low gravity. J. Appl. Physiol. 71, 863–870. 10.1152/jappl.1991.71.3.863 1757322

[B24] Kipp S. Grabowski A. M. Kram R. (2018). What determines the metabolic cost of human running across a wide range of velocities? J. Exp. Biol. 221:184218 10.1242/jeb.184218 30065039

[B25] Kram R. Taylor C. R. (1990). Energetics of running: a new perspective. Nature 346, 265–267. 10.1038/346265a0 2374590

[B26] Lacour J. R. Bourdin M. (2015). Factors affecting the energy cost of level running at submaximal speed. Eur. J. Appl. Physiol. 115, 651–673. 10.1007/s00421-015-3115-y 25681108

[B27] Lai A. Schache A. G. Lin Y. C. Pandy M. G. (2014). Tendon elastic strain energy in the human ankle plantar-flexors and its role with increased running speed. J. Exp. Biol. 217(Pt 17), 3159–3168. 10.1242/jeb.100826 24948642

[B28] McMahon T. A. Cheng G. C. (1990). The mechanics of running: how does stiffness couple with speed? J. Biomech. 23(Suppl. 1), 65–78. 2081746 10.1016/0021-9290(90)90042-2

[B29] Minetti A. E. Ardigo L. P. Saibene F. (1994). Mechanical determinants of the minimum energy cost of gradient running in humans. J. Exp. Biol. 195, 211–225. 7964412 10.1242/jeb.195.1.211

[B30] Morin J. B. Dalleau G. Kyrolainen H. Jeannin T. Belli A. (2005). A simple method for measuring stiffness during running. J. Appl. Biomech. 21, 167–180. 10.1123/jab.21.2.167 16082017

[B31] Nardello F. Ardigo L. P. Minetti A. E. (2011). Measured and predicted mechanical internal work in human locomotion. Hum. Mov. Sci. 30, 90–104. 10.1016/j.humov.2010.05.012 21056491

[B32] Nummela A. Keranen T. Mikkelsson L. O. (2007). Factors related to top running speed and economy. Int. J. Sports Med. 28, 655–661. 10.1055/s-2007-964896 17549657

[B33] Pavei G. Biancardi C. M. Minetti A. E. (2015). Skipping vs. running as the bipedal gait of choice in hypogravity. J. Appl. Physiol. 119, 93–100. 10.1152/japplphysiol.01021.2014 25930029

[B34] Raffalt P. C. Hovgaard-Hansen L. Jensen B. R. (2013). Running on a lower-body positive pressure treadmill: VO2max, respiratory response, and vertical ground reaction force. Res. Q. Exerc. Sport 84, 213–222. 10.1080/02701367.2013.784721 23930547

[B35] Rathkey J. K. Wall-Scheffler C. M. (2017). People choose to run at their optimal speed. Am. J. Phys. Anthropol. 163, 85–93. 10.1002/ajpa.23187 28195301

[B36] Saibene F. Minetti A. E. (2003). Biomechanical and physiological aspects of legged locomotion in humans. Eur. J. Appl. Physiol. 88, 297–316. 10.1007/s00421-002-0654-9 12527959

[B37] Sainton P. Nicol C. Cabri J. Barthelemy-Montfort J. Berton E. Chavet P. (2015). Influence of short-term unweighing and reloading on running kinetics and muscle activity. Eur. J. Appl. Physiol. 115, 1135–1145. 10.1007/s00421-014-3095-3 25566954

[B38] Sasaki K. Neptune R. R. (2006). Muscle mechanical work and elastic energy utilization during walking and running near the preferred gait transition speed. Gait Posture 23, 383–390. 10.1016/j.gaitpost.2005.05.002 16029949

[B39] Seyfarth A. Geyer H. Gunther M. Blickhan R. (2002). A movement criterion for running. J. Biomech. 35, 649–655. 10.1016/S0021-9290(01)00245-7 11955504

[B40] Shaw A. J. Ingham S. A. Folland J. P. (2014). The valid measurement of running economy in runners. Med. Sci. Sports Exerc. 46, 1968–1973. 10.1249/MSS.0000000000000311 24561819

[B41] Shaw A. J. Ingham S. A. Fudge B. W. Folland J. P. (2013). The reliability of running economy expressed as oxygen cost and energy cost in trained distance runners. Appl. Physiol. Nutr. Metab. 38, 1268–1272. 10.1139/apnm-2013-0055 24195628

[B42] Srinivasan M. (2009). Optimal speeds for walking and running, and walking on a moving walkway. Chaos 19:026112 10.1063/1.3141428 19566272

[B43] Stainsby W. N. Barclay J. K. (1970). Exercise metabolism: O 2 deficit, steady level O 2 uptake and O 2 uptake for recovery. Med. Sci. Sports 2, 177–181. 5521271

[B44] Steudel-Numbers K. L. Wall-Scheffler C. M. (2009). Optimal running speed and the evolution of hominin hunting strategies. J. Hum. Evol. 56, 355–360. 10.1016/j.jhevol.2008.11.002 19297009

[B45] Steudel-Numbers K. L. Weaver T. D. Wall-Scheffler C. M. (2007). The evolution of human running: effects of changes in lower-limb length on locomotor economy. J. Hum. Evol. 53, 191–196. 10.1016/j.jhevol.2007.04.001 17574650

[B46] Taylor C. R. (1985). Force development during sustained locomotion: a determinant of gait, speed and metabolic power. J. Exp. Biol. 115, 253–262. 4031768 10.1242/jeb.115.1.253

[B47] Teunissen L. P. Grabowski A. Kram R. (2007). Effects of independently altering body weight and body mass on the metabolic cost of running. J. Exp. Biol. 210(Pt 24), 4418–4427. 10.1242/jeb.004481 18055630

[B48] Wiesinger H. P. Rieder F. Kosters A. Muller E. Seynnes O. R. (2016). Are sport-specific profiles of tendon stiffness and cross-sectional area determined by structural or functional integrity? PLoS ONE 11:e0158441 10.1371/journal.pone.0158441 27362657 PMC4928785

[B49] Wiesinger H. P. Rieder F. Kosters A. Muller E. Seynnes O. R. (2017). Sport-specific capacity to use elastic energy in the patellar and achilles tendons of elite athletes. Front. Physiol. 8:132. 10.3389/fphys.2017.00132 28348529 PMC5346584

[B50] Willcockson M. A. Wall-Scheffler C. M. (2012). Reconsidering the effects of respiratory constraints on the optimal running speed. Med. Sci. Sports Exerc. 44, 1344–1350. 10.1249/MSS.0b013e318248d907 22217570

[B51] Willems P. A. Cavagna G. A. Heglund N. C. (1995). External, internal and total work in human locomotion. J. Exp. Biol. 198(Pt 2), 379–393. 7699313 10.1242/jeb.198.2.379

