# Supplementary material for: Mechanical Determinants of the U-Shaped Speed-Energy Cost of Running Relationship
Source: Front Physiol. 2018 Dec 18;9:1790. doi: 10.3389/fphys.2018.01790 (PMC6305502; doi:10.3389/fphys.2018.01790)
Supplement: Supplementary file 1 [file Data_Sheet_1.docx]

**ADDITIONAL FILE**

**Mechanical determinants of the U-shaped speed-energy cost of running relationship**

Apolline Carrard^1,§^, Elisa Fontana^1,§^, Davide Malatesta^1,*^

^1^Institute of Sport Sciences (ISSUL), Faculty of Biology and Medicine, University of Lausanne, Lausanne, Switzerland.

**Running title:** Mechanical determinants of energy cost of running.

**^*^Corresponding author**

Davide Malatesta

University of Lausanne (UNIL), Institute of Sport Sciences of University of Lausanne, Bâtiment Synathlon, 1015 Lausanne, Switzerland.

Telephone: +41 21 692 36 17 - Fax: +41 (0) 21 692 32 93

E-Mail: davide.malatesta@unil.ch

ORCID: 0000-0003-3905-5642

^§^These authors contributed equally to this work.

**Results**

*Net energy cost per kg of body mass.* The two-way RM ANOVA revealed a main effect of gravity (P < 0.001); the net Cr was significantly different at all measured %BW (Figure 1A). A significant main speed effect (P = 0.004) was found, suggesting that the net Cr changed with running speeds for the 3 gravity conditions (Figure 1A). This statistically confirmed the AIC system results attesting that the curvilinear modeling better fit the speed-Cr relationship than the linear model (see below). The speed x gravity interaction effect was also significant (P = 0.014) (Figure 1A). At 100% BW, the net Cr was significantly greater at 5.00 than at 3.17 and 4.08 m∙s^-1^ (P ≤ 0.02) and tended to be higher at 2.25 than at 3.17 m∙s^-1^ (P = 0.073). At 60% BW, the net Cr was significantly greater at 2.25 than at 3.17 m∙s^-1^ (P = 0.02) and tended to be higher at 2.25 than at 4.08 m∙s^-1^ (P = 0.09). At 20% BW, the net Cr was significantly greater at 2.25 than at 3.17 and 4.08 m∙s^-1^ (P ≤ 0.047).

*Net energy cost per body weight.* The two-way RM ANOVA revealed a main effect of gravity (P < 0.001); the net Cr normalized to BW was significantly different at all measured %BW (Figure 1B). Significant main speed effect was also found (P = 0.018) with no significant speed x gravity interaction effect (P = 0.136; Figure 1B). The net Cr was significantly greater at 2.25 than at 3.17 and 4.08 m∙s^-1^ (P ≤ 0.017).

*Linear and curvilinear models for the speed- net Cr per kg of body mass relationship*. For the speed-Cr relationship, there was a negative ΔAIC, suggesting that the curvilinear model provided a better fit than the linear model for the 3 conditions (ΔAIC equation: -3.9 ± 5.7, -5.2 ± 6.7 and -6.9 ± 8.0 at 20%, 60% and 100% BW, respectively). The mean r^2^ for fitting a curvilinear model to the speed-Cr relationship across all subjects was 0.73 ± 0.28, 0.81 ± 0.22 and 0.85 ± 0.21 at 20%, 60% and 100% BW, respectively, while the mean r^2^ with the linear model was only 0.37 ± 0.31, 0.40 ± 0.31 and 0.38 ± 0.26 at 20%, 60% and 100% BW, respectively.

**Figure caption**

**Figure 1.** Net energy cost per kg of body mass (A) and net energy cost per body weight (B) versus running speed at 100% body weight (BW; 1 *g*), 60% BW (0.6 *g*) and 20% BW (0.2 *g*) (n = 12). Values are mean ± SD. *P < 0.05 for the significant speed effect; †P < 0.05 for the significant gravity effect; ‡P < 0.05 for the significant interaction effect; *2 for significant difference from 3.17 m∙s^-1^; *3 for significant difference from 4.08 m∙s^-1^; and *4 for significant difference from 5.0 m∙s^-1^ (P < 0.05). §2 for tendency from 3.17 m∙s^-1^; §3 for tendency from 4.08 m∙s^-1^ (P < 0.1). There was a significant gravity effect for each speed (P < 0.001; for sake of clarity, these significant differences are not shown).

| 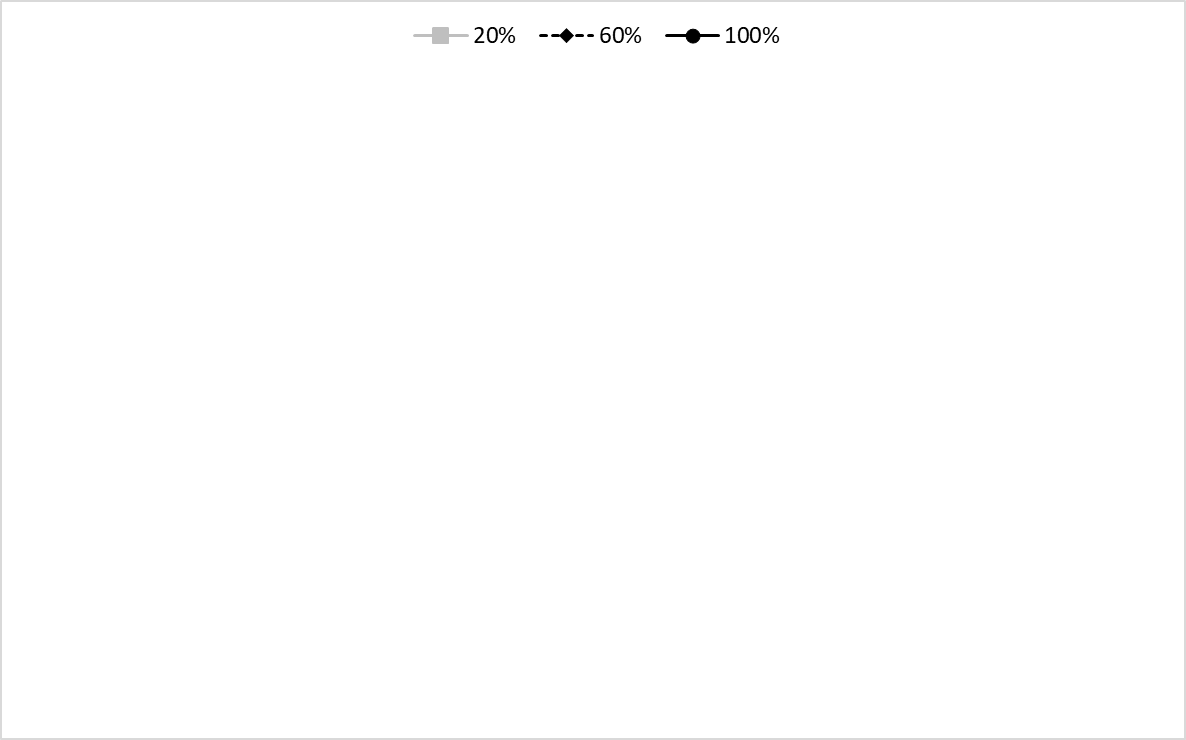 |
| --- |
| **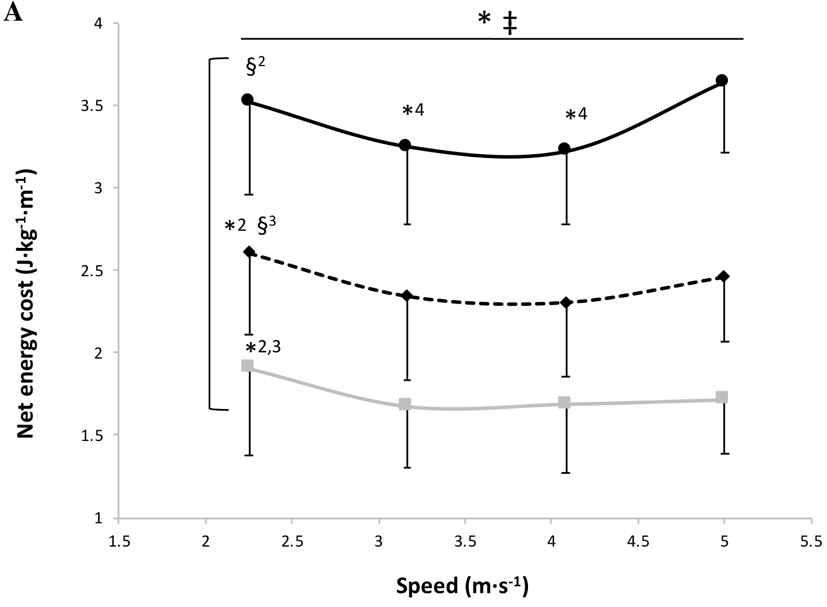** |
| **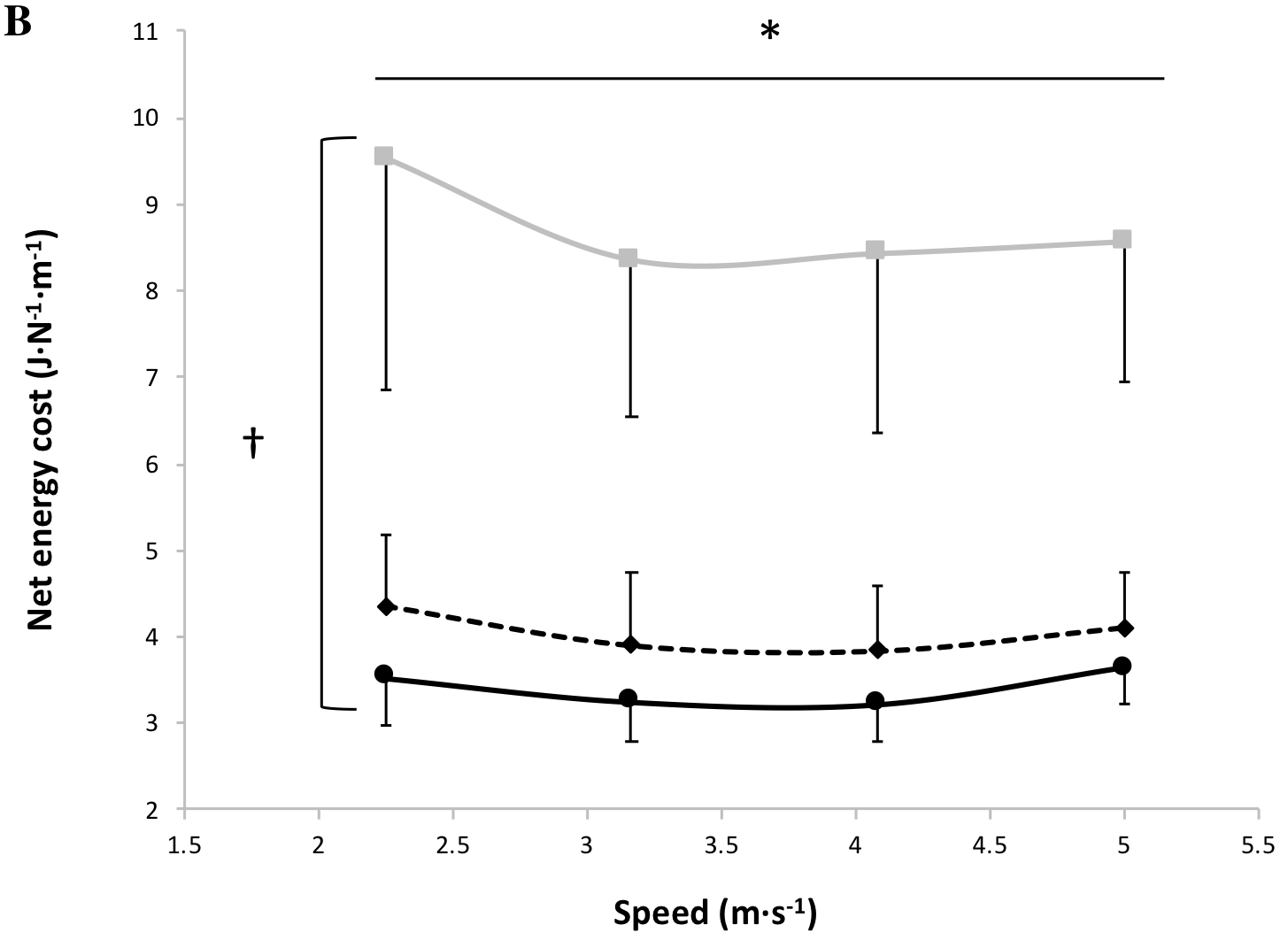** |
|  |

**Figure 1**
